# Actin fence therapy with exogenous V12Rac1 protects against acute lung injury

**DOI:** 10.1172/jci.insight.150137

**Published:** 2021-04-22

**Authors:** Galina A. Gusarova, Shonit R. Das, Mohammad N. Islam, Kristin Westphalen, Guangchun Jin, Igor O. Shmarakov, Li Li, Sunita Bhattacharya, Jahar Bhattacharya

Original citation: *JCI Insight.* 2021;6(6):e135753. https://doi.org/10.1172/jci.insight.135753

Citation for this corrigendum: *JCI Insight*. 2021;6(8):e150137. https://doi.org/10.1172/jci.insight.150137

During the preparation of this manuscript, the center panel of the bottom row of Figure 2D was inadvertently duplicated from the left panel of the bottom row of Figure 2D. The figure has been corrected.

The authors regret the error.

## Version 1 04/22/2021

Electronic publication

## Version 2 05/19/2021

Electronic publication.

