# Supplementary material for: Actin fence therapy with exogenous V12Rac1 protects against acute lung injury
Source: JCI Insight. 2021 Mar 22;6(6):e135753. doi: 10.1172/jci.insight.135753 (PMC8026177; doi:10.1172/jci.insight.135753)
Supplement: Supplemental data [file jciinsight-6-135753-s228.pdf]

Supplementary Materials for

Actin fence therapy with exogenous V12Rac1 protects against Acute Lung Injury

Authors: Galina A. Gusarova, Shonit R. Das, Mohammad N. Islam, Kristin Westphalen, Guangchun Jin, Igor O. Shmarakov, Li Li, Sunita Bhattacharya, Jahar Bhattacharya*

*Corresponding author. E-mail: jb39@cumc.columbia.edu (JB)

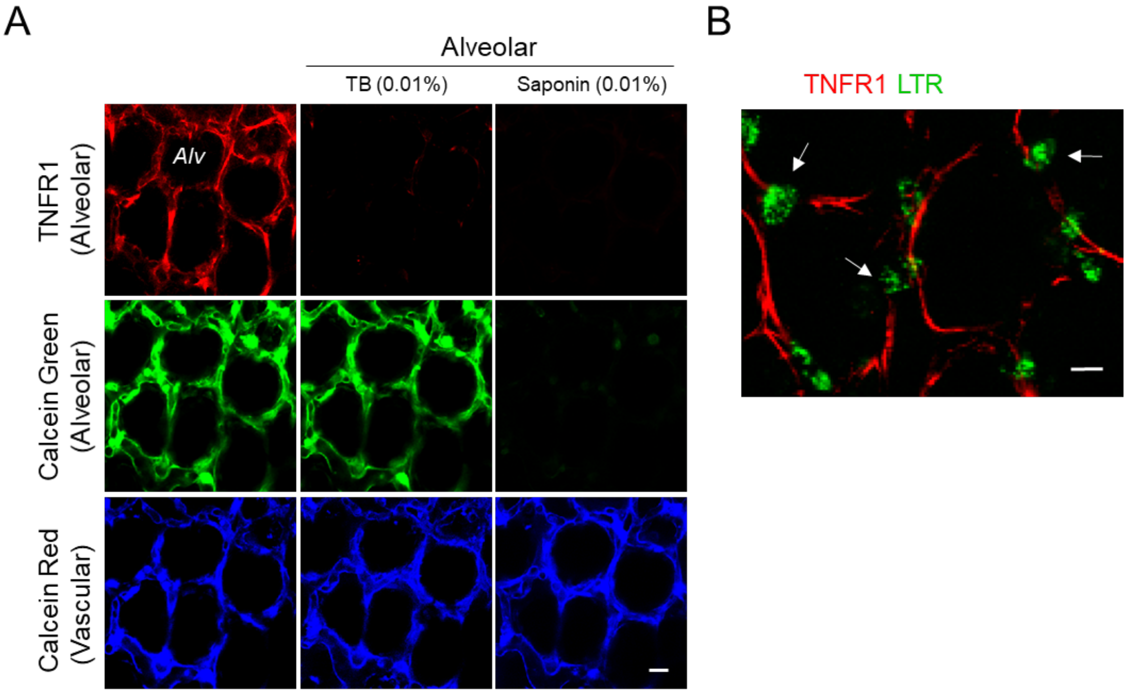

Supplemental Figure 1. Alveolar expression of TNFR1. (A) Confocal images show live alveolar epithelial (top and middle row) and subjacent vascular endothelial fluorescence (bottom row) at baseline (first column), and following trypan blue (second column) and saponin (third column) treatments. In the top and middle panels, alveolar microinjections of anti-TNFR1 mAb and calcein green label the same alveoli. The bottom panel shows microvascular endothelium loaded with vascular infusion of calcein red (blue pseudocolor). Alveoli were microinfused with the membrane-impermeable fluorescence quencher trypan blue (TB) and the membrane permeabilizing agent, saponin as indicated. Note, that TB abolished TNFR1 fluorescence, indicating that the immunofluorescence was on the epithelial surface, and saponin caused fluorescence loss of epithelial, but not of endothelial cytosolic fluorescence, indicating that alveolar injections targeted only the alveolar epithelium. (**B**) Live image of alveoli shows fluorescence of TNFR1 (red). AT2 cells (*arrows*) are identified by Lysotracker fluorescence (green). Note, AT2 cells do not express TNFR1 fluorescence. Scale bar, 10 µm. Replicated in 5 lungs.

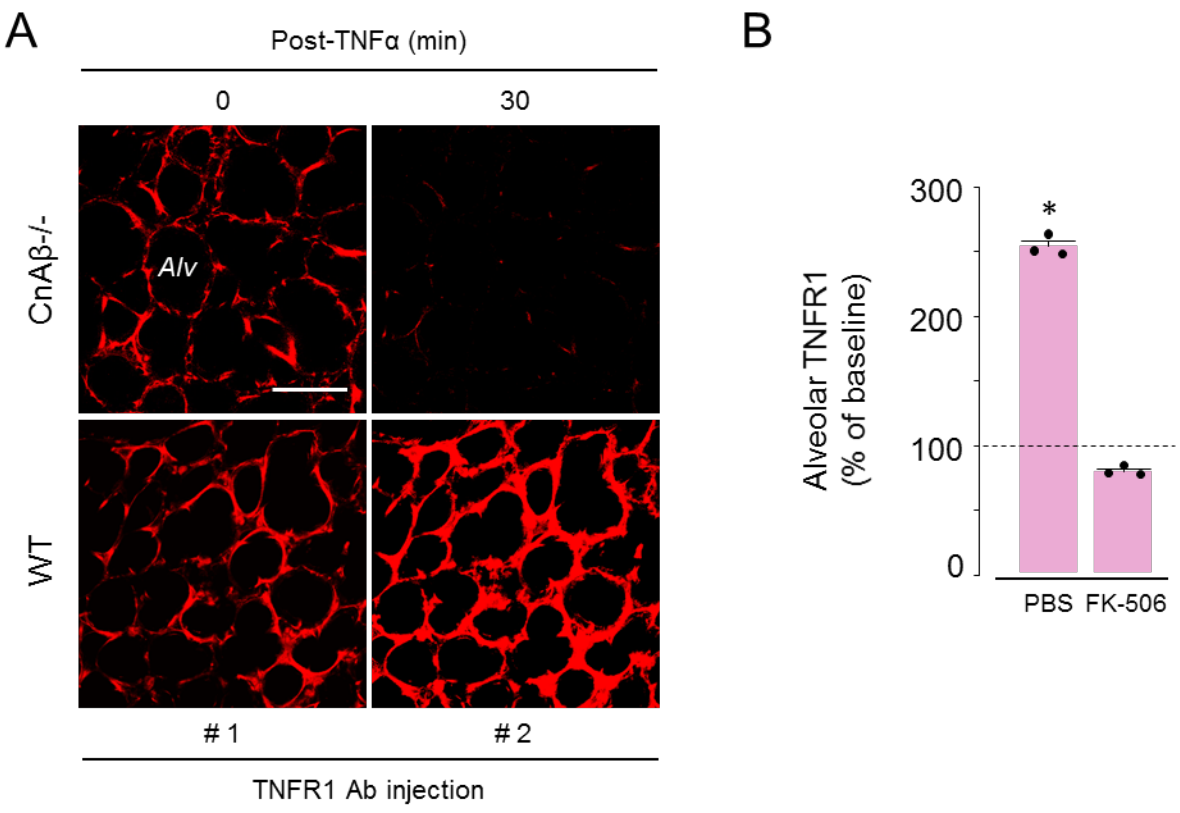

Supplemental Figure 2. Calcineurin determines alveolar TNFR1 expression. (**A**) Confocal images show alveolar TNFR1 expression before and after alveolar TNFα microinjection in wild type (*WT*) and calcineurin-Aβ null (*CnAβ-/-*) mice. Note, 30-min images show recovery of TNFR1 expression in *WT* but not in *CnAβ* -/-. *Alv*, alveolus. Scale bars, 50 µm. Replicated in 3 lungs. (**B**) Data were obtained as whole-image fluorescence at baseline (*dashed line*) and 30 min after alveolar microinfusion of TNFα in lungs pre-treated with *FK-506*. Microinjections of anti-TNFR1 Ab for TNFR1 detection were given prior to and 30 min after TNFα. Mean±SE, n=3 lungs for each group, *$p<0.05$ vs baseline using 2-tailed t test. Each dot shows data for a single lung.

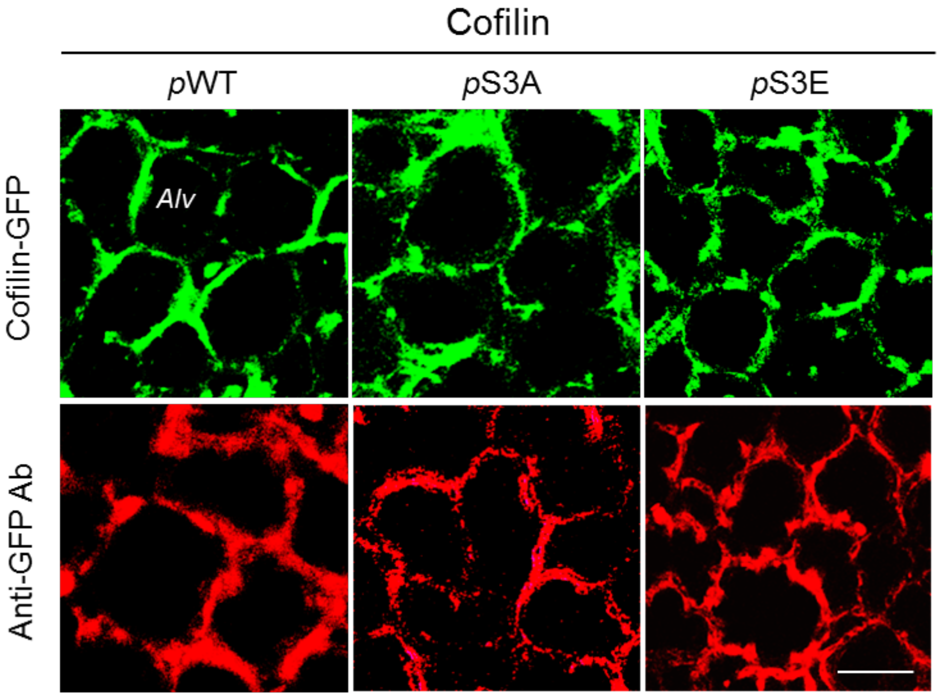

Supplemental Figure 3. Expression of cofilin mutants in alveoli. Confocal images show fluorescence of the expressed GFP-cofilin mutants (green) and anti-GFP immunofluorescence (red) confirming alveolar expression of the cofilin. Cofilin transfections were for wild-type plasmid (*p*WT), and for constitutively active (*p*S3A), or inactive (*p*S3E) cofilin mutants; *Alv*, alveolus. Scale bars, 50 μm. Replicated in 3 lungs.

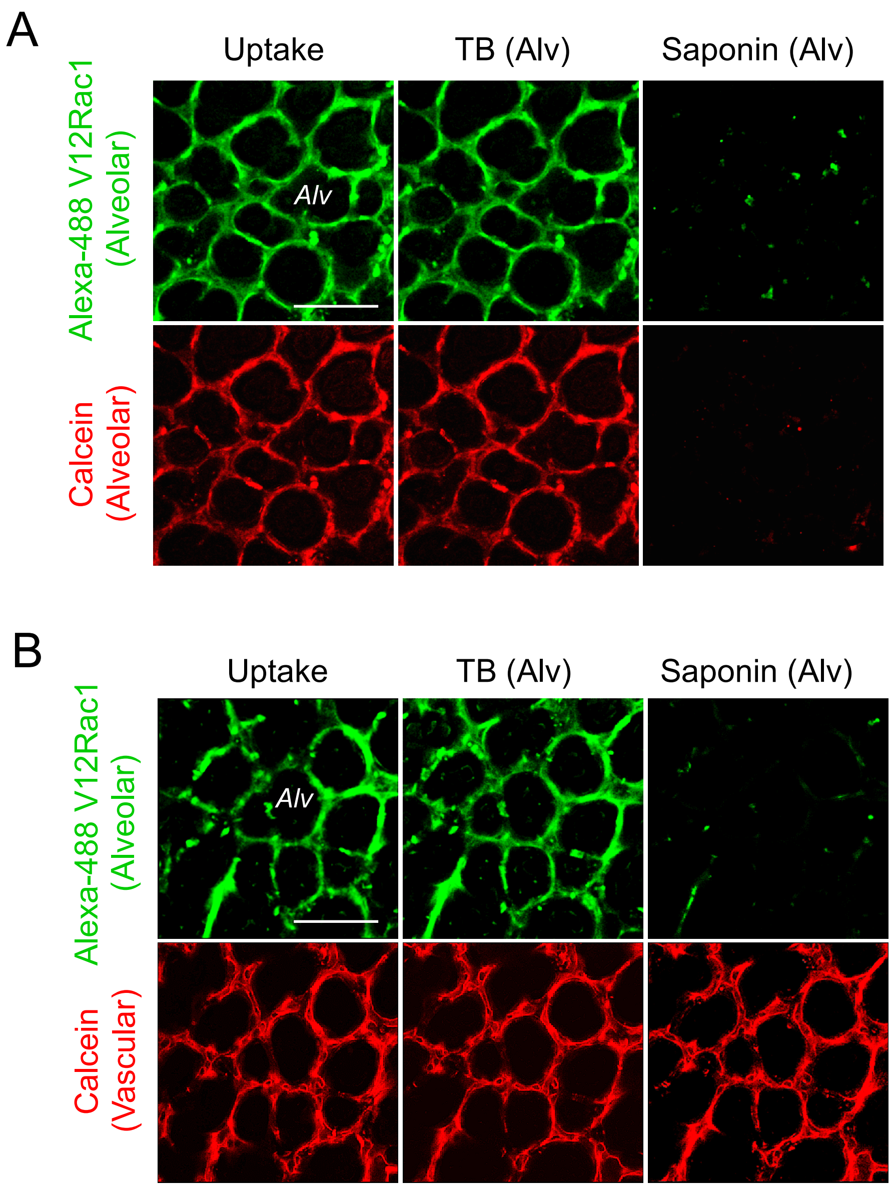

Supplemental Figure 4. Rac1 uptake by alveolar epithelium. Confocal images show epithelial internalization of TAT-conjugated V12 Rac1 (upper panels, green) (**A, B**). Cytosolic dye, calcein (red) delineates the alveolar epithelium (**A**) and vascular endothelium (**B**). Alveolar infusion of trypan blue (TB) did not quench green fluorescence, confirming V12 Rac1 was internalized. Alveolar infusion of saponin decreased calcein fluorescence in alveoli (**A**), but not in microvessels (**B**), indicating V12 Rac1 was internalized by alveolar epithelium alone. *Alv*, alveolus. Scale bars, 50 μm. Replicated in 2 lungs.

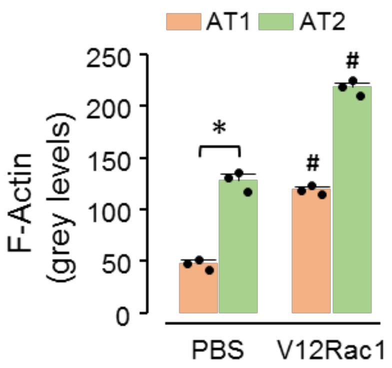

Supplemental Figure 5. Effect of Rac1 on alveolar epithelial cells. Bars show fluorescence quantifications of AT1 and AT2 actin after the indicated treatments. Each bar is mean±SE for 8 quantifications per lung, in 3 lungs. *$p < 0.05$ as indicated, #$p < 0.05$ versus corresponding baseline using 2 tailed *t* test. The dots are means of determinations for each lung.

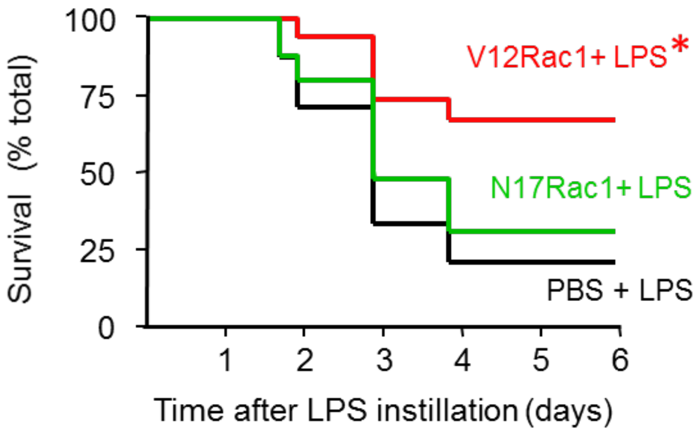

Supplemental Figure 6. Mouse survival. Kaplan-Meier plots show survival in mice pre-treated i.n. with TAT-Rac proteins, followed 30 mins later with intranasal LPS (LD80) instillation. n=15 mice in each group, *$p<0.05$ versus LPS using long-rank test.

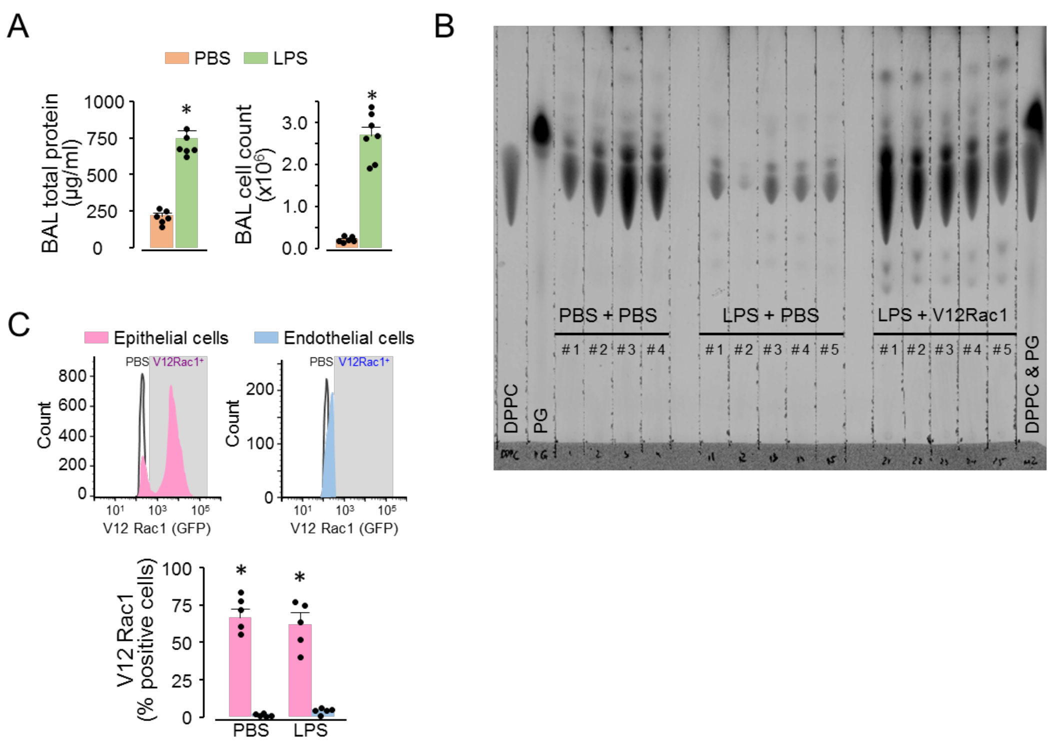

Supplemental Figure 7. Global Rac1 uptake and Thin-Layer Chromatography (TLC) analysis of BAL.

(A-C) Mice were given intranasal instillation of LPS at LD80 or PBS. **A** shows BAL total protein and leukocyte counts 4 h after LPS. Mean±SE, *p<0.05 vs PBS using 2-tailed t test. n=6 in PBS, and n=7 in LPS groups. Each dot shows data for a single lung. In **B** and **C**, intranasal TAT-V12Rac1 was given 4h after the LPS or PBS instillations, then after 72 (**B**) or 1 (**C**) hour, lungs were removed for BAL phospholipid and flow cytometry analyses, respectively. **B** shows an image of a TLC plate for phospholipid analyses. n=5, except PBS (n=4). Histograms in **C** show fluorescent V12Rac1 uptake (grey box). Bars are percentages of V12Rac1-positive cells in the indicated cell types. Mean±SE, n=5 lungs for each group, *p<0.05 using ANOVA with Bonferroni correction. Each dot shows data for a single lung.

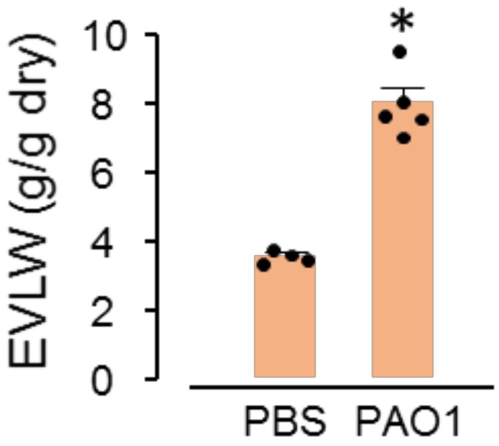

Supplemental Figure 8. Quantification of pulmonary edema. Bars show blood-free extravascular lung water (EVLW) determined 4 h after *i.n.* instillation of *P. aeruginosa* (PAO1) or PBS. Mean±SE, n=4 for PBS and n=5 for PAO1. *p<0.05 *vs*. PBS using 2-tailed t test. Each dot shows data for a single lung.
